# Supplementary material for: Artificial intelligence and leukocyte epigenomics: Evaluation and prediction of late-onset Alzheimer’s disease
Source: PLoS One. 2021 Mar 31;16(3):e0248375. doi: 10.1371/journal.pone.0248375 (PMC8011726; doi:10.1371/journal.pone.0248375)
Supplement: S1 Table — (DOCX) [file pone.0248375.s001.docx]

**Supplemental Table S1:** Clinical and demographic characteristics: AD compared to unaffected control subjects

|  | **Mean (SD)** | | **p-value** |
| --- | --- | --- | --- |
|  | **Cases** | **Controls** |  |
| **Number** | 24 | 24 | - |
| **Age (SD)** | 83.17 (7.97) | 80.04 (8.42) | 0.17 |
| **Gender (n)** | | | |
| - Female | 17 (70.83%) | 16 (66.66%) | 0.53 |
| - Male | 7 (29.16%) | 8 (33.33%) |  |
| **Race** | | | |
| - White | 18 (75%) | 16 (66.66%) | 0.49 |
| - African American | 4 (16.66%) | 4 (16.66%) |  |
| - Unknown | 2 (8.33%) | 4 (16.66%) |  |
| ***MMSE Score (mean /SD)** | 16.69 (8.55%) | 29.08 (1.04%) | 1.54x10^-7^ |
| **Hyperlipidemia** | | | |
| - Yes | 9 (37.5%) | 14 (58.3%) | 0.15 |
| - No | 15 (62.5%) | 10 (41.7%) |  |
| **Hypertension** | | | |
| - Yes | 15 (62.5%) | 17 (70.83%) | 0.76 |
| - No | 9 (37.5%) | 7 (29.16%) |  |
| **Stroke** | | | |
| - Yes | 1 (4.16%) | 2 (8.33%) | 0.57 |
| - No | 23 (95.83%) | 22 (91.66%) |  |
| **Depression** | | | |
| - Yes | 10 (41.7%) | 6 (25%) | 0.12 |
| - No | 14 (58.3%) | 18 (75%) |  |

* p-value was calculated by taking 23 samples from each group into account as one sample from each group had missing MMSE score.
