# Supplementary material for: Artificial intelligence and leukocyte epigenomics: Evaluation and prediction of late-onset Alzheimer’s disease
Source: PLoS One. 2021 Mar 31;16(3):e0248375. doi: 10.1371/journal.pone.0248375 (PMC8011726; doi:10.1371/journal.pone.0248375)
Supplement: S2 Table — (DOCX) [file pone.0248375.s002.docx]

**Supplemental Table S2:** Remaining (119 among 152) differentially methylated significant intragenic CpG markers

| **Target ID** | **CHR** | **Gene** | **FDR p-Val** | **Fold  change** | **AUC** | **CI** | | **% Methylation** | | **% Methylation difference** |
| --- | --- | --- | --- | --- | --- | --- | --- | --- | --- | --- |
|  |  |  |  |  |  | **Lower** | **Upper** | **Cases** | **Control** |  |
| cg02282594 | 14 | PRMT5;  LOC101926933 | 6.65559E-07 | 0.64 | 0.59 | 0.43 | 0.75 | 10.65 | 16.59 | -5.94 |
| cg20474604 | 9 | PTPRD | 7.89683E-07 | 0.66 | 0.68 | 0.53 | 0.83 | 11.78 | 17.91 | -6.13 |
| cg03352287 | 1 | CRCT1 | 1.12951E-06 | 1.70 | 0.74 | 0.60 | 0.88 | 11.59 | 6.81 | 4.78 |
| cg10713626 | 1 | TXNIP | 2.66128E-06 | 1.51 | 0.71 | 0.57 | 0.86 | 15.82 | 10.47 | 5.35 |
| cg02003233 | 6 | ZSCAN26 | 4.41077E-06 | 1.59 | 0.67 | 0.52 | 0.82 | 12.93 | 8.11 | 4.82 |
| cg11099754 | 10 | MPP7 | 1.4294E-05 | 0.61 | 0.73 | 0.58 | 0.87 | 7.26 | 11.89 | -4.63 |
| cg05724200 | 6 | TSTD3 | 1.67816E-05 | 0.40 | 0.60 | 0.43 | 0.76 | 2.37 | 5.90 | -3.53 |
| cg07664000 | 17 | TMIGD1 | 1.79633E-05 | 0.66 | 0.74 | 0.59 | 0.88 | 9.78 | 14.87 | -5.09 |
| cg22888958 | 7 | CREB5 | 2.59495E-05 | 1.63 | 0.62 | 0.46 | 0.78 | 10.91 | 6.68 | 4.24 |
| cg14223866 | 2 | CPS1;LANCL1 | 2.62469E-05 | 0.62 | 0.62 | 0.46 | 0.78 | 7.39 | 11.93 | -4.54 |
| cg19533489 | 16 | ZCCHC14;  LOC101928737 | 2.76668E-05 | 1.69 | 0.67 | 0.51 | 0.82 | 10.02 | 5.94 | 4.08 |
| cg12564182 | 8 | NRG1 | 3.56709E-05 | 1.51 | 0.65 | 0.49 | 0.80 | 13.78 | 9.16 | 4.63 |
| cg20934893 | 16 | RBFOX1 | 3.69582E-05 | 1.73 | 0.65 | 0.49 | 0.80 | 9.27 | 5.36 | 3.92 |
| cg25224852 | 7 | KCNH2 | 5.39805E-05 | 0.40 | 0.70 | 0.55 | 0.85 | 2.20 | 5.50 | -3.31 |
| cg15864601 | 17 | C17orf97 | 5.77271E-05 | 0.66 | 0.56 | 0.40 | 0.72 | 8.94 | 13.62 | -4.68 |
| cg01616519 | 1 | SVBP;ERMAP | 7.18189E-05 | 0.58 | 0.73 | 0.58 | 0.87 | 5.60 | 9.59 | -3.99 |
| cg01351822 | 15 | UNC45A | 9.51459E-05 | 1.61 | 0.54 | 0.38 | 0.71 | 10.42 | 6.46 | 3.96 |
| cg09826622 | 2 | FAM124B | 0.000106266 | 1.50 | 0.70 | 0.55 | 0.85 | 12.89 | 8.58 | 4.31 |
| cg04770550 | 19 | VAV1 | 0.000108068 | 1.69 | 0.68 | 0.52 | 0.83 | 9.14 | 5.40 | 3.74 |
| cg13548034 | 13 | COG6 | 0.000135246 | 1.56 | 0.57 | 0.41 | 0.73 | 11.13 | 7.11 | 4.01 |
| cg03316672 | 4 | NDUFC1;  NAA15 | 0.000309685 | 0.66 | 0.74 | 0.60 | 0.88 | 8.03 | 12.18 | -4.15 |
| cg08067721 | 17 | WNT9B | 0.000341115 | 2.35 | 0.65 | 0.49 | 0.81 | 5.11 | 2.17 | 2.93 |
| cg22852879 | 5 | RBM27 | 0.000414754 | 1.66 | 0.74 | 0.60 | 0.88 | 8.73 | 5.26 | 3.47 |
| cg16001418 | 19 | HCST | 0.000430118 | 1.62 | 0.68 | 0.52 | 0.83 | 9.25 | 5.70 | 3.55 |
| cg25433188 | 10 | SFMBT2 | 0.000537554 | 1.79 | 0.72 | 0.57 | 0.86 | 7.31 | 4.09 | 3.22 |
| cg15099643 | 1 | ERO1LB | 0.00062713 | 1.57 | 0.81 | 0.69 | 0.93 | 9.75 | 6.19 | 3.56 |
| cg14249348 | 4 | ZAR1 | 0.000645506 | 1.52 | 0.63 | 0.47 | 0.79 | 10.78 | 7.08 | 3.70 |
| ch.20.1436892F | 20 | SS18L1 | 0.000674455 | 1.55 | 0.68 | 0.53 | 0.83 | 10.22 | 6.61 | 3.61 |
| cg21123573 | 12 | SIRT4 | 0.000700645 | 0.65 | 0.73 | 0.58 | 0.87 | 7.04 | 10.84 | -3.79 |
| cg16898124 | 3 | ZXDC | 0.000941346 | 1.73 | 0.60 | 0.44 | 0.76 | 7.53 | 4.36 | 3.17 |
| cg12740438 | 5 | CTD-2194D22.4;  IRX4 | 0.001010019 | 1.60 | 0.63 | 0.47 | 0.78 | 9.02 | 5.65 | 3.37 |
| cg11585769 | 9 | CTSV | 0.001213165 | 1.63 | 0.64 | 0.48 | 0.79 | 8.36 | 5.11 | 3.24 |
| cg15167008 | 10 | CCDC3 | 0.001305305 | 1.89 | 0.63 | 0.47 | 0.79 | 6.18 | 3.27 | 2.91 |
| cg22729335 | 19 | TIMM13 | 0.001423416 | 0.58 | 0.51 | 0.35 | 0.68 | 4.39 | 7.57 | -3.17 |
| cg06693277 | 6 | GTF2H4;  VARS2 | 0.001692303 | 2.02 | 0.61 | 0.45 | 0.77 | 5.50 | 2.73 | 2.77 |
| cg09799983 | 2 | CYP1B1 | 0.0017084 | 1.61 | 0.69 | 0.54 | 0.84 | 8.50 | 5.30 | 3.21 |
| ch.1.2648414R | 1 | ST7L | 0.001997576 | 0.65 | 0.73 | 0.59 | 0.87 | 6.28 | 9.70 | -3.43 |
| cg22333960 | 1 | IGSF21 | 0.002051178 | 1.50 | 0.60 | 0.44 | 0.76 | 10.16 | 6.76 | 3.40 |
| cg25591794 | 13 | PRHOXNB | 0.002184182 | 0.60 | 0.52 | 0.36 | 0.69 | 4.69 | 7.83 | -3.14 |
| cg18608498 | 10 | ATAD1;CFLP1 | 0.002458547 | 0.60 | 0.77 | 0.63 | 0.90 | 4.54 | 7.63 | -3.09 |
| cg12700788 | 17 | KIF19 | 0.002767436 | 1.85 | 0.74 | 0.60 | 0.88 | 6.01 | 3.24 | 2.77 |
| cg18708365 | 7 | ORC5L | 0.002878528 | 0.61 | 0.62 | 0.46 | 0.78 | 4.99 | 8.12 | -3.13 |
| cg03536474 | 12 | HOXC11 | 0.00349354 | 1.56 | 0.68 | 0.52 | 0.83 | 8.61 | 5.52 | 3.09 |
| cg16308461 | 17 | CFAP52;STX8 | 0.003589552 | 0.60 | 0.71 | 0.56 | 0.86 | 4.55 | 7.56 | -3.01 |
| cg01275588 | 17 | TMEM220 | 0.003863131 | 1.58 | 0.62 | 0.46 | 0.78 | 8.21 | 5.19 | 3.02 |
| cg24533989 | 4 | ATP8A1 | 0.003912142 | 0.49 | 0.57 | 0.41 | 0.73 | 2.59 | 5.24 | -2.65 |
| cg19126300 | 11 | WT1 | 0.004057848 | 1.90 | 0.66 | 0.51 | 0.81 | 5.60 | 2.95 | 2.65 |
| cg25323545 | 15 | UBE2Q2P2;  GOLGA6L17P;  GOLGA6L9 | 0.004342098 | 1.56 | 0.59 | 0.42 | 0.75 | 8.48 | 5.45 | 3.03 |
| cg23882658 | 17 | HIC1 | 0.004343704 | 1.59 | 0.67 | 0.52 | 0.83 | 8.01 | 5.04 | 2.97 |
| cg03150279 | 9 | SCAI | 0.004415513 | 1.50 | 0.64 | 0.48 | 0.79 | 9.44 | 6.28 | 3.16 |
| cg05309948 | 11 | ASCL2 | 0.004955262 | 1.65 | 0.69 | 0.54 | 0.84 | 7.19 | 4.35 | 2.83 |
| cg13774792 | 11 | IGSF9B | 0.005214641 | 1.57 | 0.74 | 0.59 | 0.88 | 8.10 | 5.16 | 2.95 |
| cg02077793 | 19 | CARM1 | 0.005391677 | 1.60 | 0.63 | 0.47 | 0.79 | 7.65 | 4.77 | 2.88 |
| cg23207361 | 6 | BAT2 | 0.005531057 | 1.59 | 0.63 | 0.47 | 0.79 | 7.81 | 4.92 | 2.90 |
| cg06386517 | 7 | GRB10 | 0.00564335 | 2.84 | 0.79 | 0.66 | 0.92 | 3.55 | 1.25 | 2.30 |
| cg21809331 | 7 | RBM28 | 0.006069078 | 0.59 | 0.80 | 0.68 | 0.93 | 3.93 | 6.72 | -2.79 |
| cg08104202 | 1 | C1orf114 | 0.007029021 | 2.03 | 0.69 | 0.54 | 0.84 | 4.83 | 2.38 | 2.45 |
| cg24348114 | 19 | USF2;LSR | 0.007261096 | 1.51 | 0.72 | 0.57 | 0.86 | 8.89 | 5.91 | 2.98 |
| cg09493505 | 7 | VWC2 | 0.007678338 | 1.59 | 0.69 | 0.54 | 0.84 | 7.54 | 4.74 | 2.80 |
| ch.15.1428207F | 15 | SEC11A | 0.007690988 | 1.64 | 0.62 | 0.46 | 0.78 | 7.00 | 4.27 | 2.73 |
| ch.14.1402140F | 14 | FOXN3 | 0.007950532 | 1.59 | 0.67 | 0.52 | 0.82 | 7.49 | 4.70 | 2.79 |
| cg12195369 | 19 | ISOC2 | 0.008047702 | 2.02 | 0.71 | 0.57 | 0.86 | 4.81 | 2.39 | 2.42 |
| cg03656099 | 17 | TBX2 | 0.008171155 | 1.72 | 0.63 | 0.47 | 0.79 | 6.28 | 3.66 | 2.62 |
| cg08668527 | 14 | TRMT61A | 0.00860437 | 1.60 | 0.55 | 0.38 | 0.71 | 7.31 | 4.57 | 2.75 |
| cg04571847 | 19 | ZNF254 | 0.009279063 | 0.48 | 0.51 | 0.34 | 0.67 | 2.25 | 4.67 | -2.42 |
| cg19529579 | 1 | LOC101928370;  S1PR1 | 0.009301792 | 1.56 | 0.72 | 0.58 | 0.87 | 7.83 | 5.03 | 2.80 |
| cg12725871 | 7 | ERV3-1 | 0.010352949 | 1.54 | 0.57 | 0.41 | 0.73 | 8.05 | 5.24 | 2.80 |
| cg00389577 | 16 | C16orf35 | 0.01057885 | 0.62 | 0.79 | 0.66 | 0.92 | 4.60 | 7.37 | -2.76 |
| cg15841063 | 1 | FOXD3 | 0.010624425 | 1.61 | 0.58 | 0.41 | 0.74 | 7.09 | 4.41 | 2.68 |
| cg11777419 | 14 | KIF26A | 0.012088628 | 2.13 | 0.68 | 0.53 | 0.83 | 4.29 | 2.01 | 2.28 |
| cg00733288 | 13 | CARS2 | 0.012218997 | 0.57 | 0.57 | 0.40 | 0.73 | 3.32 | 5.85 | -2.53 |
| cg06463365 | 1 | TAL1 | 0.012232212 | 1.56 | 0.76 | 0.62 | 0.90 | 7.58 | 4.87 | 2.71 |
| cg15758178 | 3 | STT3B | 0.012239894 | 1.50 | 0.67 | 0.52 | 0.83 | 8.39 | 5.58 | 2.81 |
| cg20842253 | 2 | TRIM54 | 0.013385148 | 0.61 | 0.64 | 0.48 | 0.80 | 4.23 | 6.88 | -2.65 |
| cg16921083 | 3 | SOX14 | 0.014056951 | 1.66 | 0.75 | 0.61 | 0.89 | 6.38 | 3.85 | 2.53 |
| cg13297865 | 6 | ELOVL4 | 0.014318167 | 2.05 | 0.67 | 0.52 | 0.82 | 4.42 | 2.15 | 2.27 |
| cg01904183 | 4 | TRAM1L1 | 0.014903863 | 0.46 | 0.76 | 0.63 | 0.90 | 1.91 | 4.18 | -2.26 |
| cg09065413 | 1 | PTCH2 | 0.016577944 | 1.62 | 0.66 | 0.50 | 0.81 | 6.61 | 4.08 | 2.52 |
| cg11199770 | 19 | TSHZ3 | 0.018202366 | 2.08 | 0.69 | 0.54 | 0.84 | 4.23 | 2.03 | 2.20 |
| cg04874239 | 12 | KRT73 | 0.018736448 | 0.61 | 0.64 | 0.48 | 0.80 | 4.02 | 6.56 | -2.54 |
| cg20447655 | 8 | CTHRC1 | 0.018985857 | 0.45 | 0.52 | 0.35 | 0.68 | 1.76 | 3.94 | -2.19 |
| cg23433828 | 16 | CRNDE | 0.019207918 | 2.13 | 0.62 | 0.46 | 0.78 | 4.10 | 1.92 | 2.17 |
| cg10200291 | 5 | FLJ44606 | 0.01960463 | 0.59 | 0.61 | 0.45 | 0.77 | 3.44 | 5.88 | -2.44 |
| ch.11.150762R | 11 | NUP98 | 0.020002555 | 0.57 | 0.68 | 0.53 | 0.83 | 3.20 | 5.59 | -2.40 |
| cg09559545 | 3 | KIAA1407;  QTRTD1 | 0.02201082 | 0.54 | 0.82 | 0.70 | 0.94 | 2.74 | 5.04 | -2.30 |
| cg19035718 | 12 | METTL20 | 0.022309736 | 0.64 | 0.66 | 0.51 | 0.82 | 4.60 | 7.17 | -2.58 |
| cg23164183 | 5 | C5orf38 | 0.022923717 | 1.71 | 0.72 | 0.57 | 0.86 | 5.59 | 3.26 | 2.33 |
| cg25864024 | 15 | FAM174B | 0.02340383 | 1.87 | 0.60 | 0.44 | 0.76 | 4.77 | 2.54 | 2.22 |
| cg13426857 | 6 | CCHCR1;TCF19 | 0.023871488 | 0.56 | 0.56 | 0.39 | 0.72 | 2.95 | 5.26 | -2.32 |
| cg07508773 | 2 | WDSUB1 | 0.024448342 | 1.58 | 0.58 | 0.42 | 0.74 | 6.64 | 4.19 | 2.45 |
| cg14604369 | 4 | SC4MOL | 0.024548879 | 0.65 | 0.65 | 0.50 | 0.81 | 4.86 | 7.45 | -2.59 |
| cg03987748 | 1 | DISC1;TSNAX | 0.027247579 | 1.54 | 0.78 | 0.64 | 0.91 | 7.11 | 4.63 | 2.48 |
| cg03457776 | 12 | PTPN6 | 0.028399463 | 1.53 | 0.61 | 0.45 | 0.77 | 7.10 | 4.63 | 2.47 |
| cg16320779 | 14 | ZFYVE1 | 0.028416626 | 1.75 | 0.72 | 0.57 | 0.86 | 5.22 | 2.98 | 2.24 |
| cg19235955 | 19 | ZNF561 | 0.031881386 | 0.59 | 0.54 | 0.37 | 0.70 | 3.32 | 5.62 | -2.30 |
| cg01792524 | 9 | CDK20 | 0.032049879 | 2.08 | 0.54 | 0.38 | 0.71 | 3.96 | 1.90 | 2.06 |
| cg02867574 | 2 | ADRA2B | 0.032516657 | 1.67 | 0.57 | 0.40 | 0.73 | 5.63 | 3.36 | 2.26 |
| cg26820055 | 2 | ARHGEF4 | 0.033538729 | 1.52 | 0.67 | 0.51 | 0.82 | 7.11 | 4.68 | 2.43 |
| cg23179168 | 8 | FAM84B | 0.033967184 | 1.60 | 0.76 | 0.63 | 0.90 | 6.19 | 3.87 | 2.32 |
| cg10044466 | 11 | ADM | 0.034578849 | 0.58 | 0.73 | 0.59 | 0.87 | 3.18 | 5.44 | -2.26 |
| cg20686234 | 17 | SARM1 | 0.035571432 | 0.41 | 0.67 | 0.51 | 0.82 | 1.40 | 3.39 | -1.99 |
| cg05435090 | 7 | HOXA7 | 0.035619416 | 1.59 | 0.71 | 0.57 | 0.86 | 6.21 | 3.90 | 2.31 |
| cg02215115 | 6 | KCNK5 | 0.036062583 | 2.18 | 0.72 | 0.57 | 0.86 | 3.69 | 1.69 | 2.00 |
| cg04016086 | 7 | COBL | 0.037466396 | 1.56 | 0.70 | 0.55 | 0.85 | 6.56 | 4.22 | 2.34 |
| cg16548177 | 1 | CLCC1 | 0.038158881 | 1.55 | 0.76 | 0.63 | 0.90 | 6.59 | 4.25 | 2.34 |
| cg18846139 | 12 | ANKS1B | 0.039282672 | 1.50 | 0.68 | 0.53 | 0.84 | 7.22 | 4.81 | 2.41 |
| cg02125316 | 5 | FGF18 | 0.039708914 | 1.52 | 0.57 | 0.41 | 0.73 | 6.94 | 4.57 | 2.37 |
| cg10505324 | 17 | HOXB8 | 0.040125935 | 1.76 | 0.75 | 0.61 | 0.89 | 4.93 | 2.80 | 2.13 |
| cg12119381 | 7 | PAXIP1-AS2 | 0.040555442 | 1.59 | 0.57 | 0.40 | 0.73 | 6.07 | 3.81 | 2.27 |
| cg23582836 | 12 | HOXC12 | 0.041000871 | 1.54 | 0.56 | 0.40 | 0.73 | 6.64 | 4.31 | 2.33 |
| cg14257369 | 19 | FGF22 | 0.043168594 | 1.87 | 0.57 | 0.41 | 0.74 | 4.43 | 2.38 | 2.06 |
| cg23726288 | 6 | PAQR8 | 0.043709823 | 1.60 | 0.78 | 0.65 | 0.91 | 5.93 | 3.70 | 2.23 |
| cg10384373 | 14 | DNAL1 | 0.04712723 | 1.52 | 0.68 | 0.53 | 0.83 | 6.69 | 4.39 | 2.30 |
| ch.4.2245532F | 4 | PRDM5 | 0.047332088 | 0.63 | 0.72 | 0.58 | 0.87 | 3.97 | 6.26 | -2.28 |
| cg07870982 | 16 | MAF | 0.047817421 | 2.33 | 0.69 | 0.54 | 0.84 | 3.33 | 1.43 | 1.90 |
| cg04363097 | 16 | PSMD7 | 0.047995996 | 1.60 | 0.76 | 0.62 | 0.90 | 5.90 | 3.69 | 2.21 |
| cg08245509 | 1 | SCYL3 | 0.048406083 | 0.66 | 0.84 | 0.72 | 0.95 | 4.56 | 6.92 | -2.36 |
| cg27051290 | 11 | SYTL2 | 0.049194471 | 1.64 | 0.73 | 0.59 | 0.88 | 5.55 | 3.39 | 2.16 |
| cg23054189 | 1 | TRIM58 | 0.049702974 | 1.60 | 0.61 | 0.45 | 0.77 | 5.88 | 3.69 | 2.20 |
