# Supplementary material for: Artificial intelligence and leukocyte epigenomics: Evaluation and prediction of late-onset Alzheimer’s disease
Source: PLoS One. 2021 Mar 31;16(3):e0248375. doi: 10.1371/journal.pone.0248375 (PMC8011726; doi:10.1371/journal.pone.0248375)
Supplement: S3 Table — (DOCX) [file pone.0248375.s003.docx]

**Supplemental Table S3**: Alzheimer’s disease prediction based on Intragenic CpG markers

*Each CpG marker in this analysis was differentially methylated in
 AD compared to controls at FDR threshold of p-value <0.05

|  | SVM | GLM | PAM | RF | LDA | DL |
| --- | --- | --- | --- | --- | --- | --- |
| AUC  95% CI | 0.9035  (0.7000-1) | 0.9067  (0.7000-1) | 0.9011  (0.7000-1) | 0.8945  (0.7000-1) | 0.8900  (0.7000-1) | 0.9150  (0.7000-1) |
| Sensitivity | 0.8100 | 0.8100 | 0.8100 | 0.8150 | 0.8100 | 0.8350 |
| Specificity | 0.8100 | 0.8100 | 0.8020 | 0.8033 | 0.8050 | 0.8300 |

Important predictors in order:

SVM: cg26677194, cg19502700, cg00352417, cg14827090, cg10596483

GLM: cg23623880, cg19502700, cg00352417, cg26677194, cg14827090

PAM: cg19502700, cg26677194, cg14827090, cg23623880, cg10596483

RF: cg23623880, cg14827090, cg14583312, cg19502700, cg13159023

LDA: cg19502700, cg10596483, cg00352417, cg23623880, cg01831771

DL: cg26677194, cg19502700, cg00352417, cg14827090, cg10596483

Support Vector Machine (SVM), Generalized Linear Model (GLM), Prediction Analysis for Microarrays (PAM), Random Forest (RF), Linear Discriminant Analysis (LDA) and Deep Learning (DL).
