# Supplementary material for: Artificial intelligence and leukocyte epigenomics: Evaluation and prediction of late-onset Alzheimer’s disease
Source: PLoS One. 2021 Mar 31;16(3):e0248375. doi: 10.1371/journal.pone.0248375 (PMC8011726; doi:10.1371/journal.pone.0248375)
Supplement: S4 Table — (DOCX) [file pone.0248375.s004.docx]

**Supplemental Table S4:** Alzheimer’s disease prediction based on Intergenic/extagenic CpG markers.

*Each CpG marker used in this analysis was differentially methylated in AD compared to controls using FDR p-value <0.05

|  | SVM | GLM | PAM | RF | LDA | DL |
| --- | --- | --- | --- | --- | --- | --- |
| AUC  95% CI | 0.9085  (0.7000-1) | 0.9111  (0.7000-1) | 0.9035  (0.7000-1) | 0.9000  (0.7000-1) | 0.8960  (0.7000-1) | 0.9190  (0.7000-1) |
| Sensitivity | 0.8000 | 0.8000 | 0.8100 | 0.8100 | 0.8100 | 0.8300 |
| Specificity | 0.8100 | 0.8000 | 0.8020 | 0.8033 | 0.8050 | 0.8300 |

Important predictors in order:

SVM: cg27055313, cg23155965, rs2208123, cg11468315, rs6626309

GLM: cg27055313, rs1941955, rs1040870, cg00224807, cg19432688

PAM: cg27055313, cg23155965, rs2032088, rs5987737, cg11468315

RF: cg27055313, cg00224807, cg11468315, rs4331560, cg23155965

LDA: cg23155965, rs2208123, cg27055313, rs5987737, cg00224807

DL: cg27055313, cg23155965, rs2208123, cg11468315, rs6626309

Support Vector Machine (SVM), Generalized Linear Model (GLM), Prediction Analysis for Microarrays (PAM), Random Forest (RF), Linear Discriminant Analysis (LDA) and Deep Learning (DL)
