# Supplementary material for: Artificial intelligence and leukocyte epigenomics: Evaluation and prediction of late-onset Alzheimer’s disease
Source: PLoS One. 2021 Mar 31;16(3):e0248375. doi: 10.1371/journal.pone.0248375 (PMC8011726; doi:10.1371/journal.pone.0248375)
Supplement: S5 Table — (DOCX) [file pone.0248375.s005.docx]

**Supplemental Table S5:** Alzheimer’s disease prediction based on Intragenic CpG markers only: Genome-wide significance threshold*

| **Parameter** | **SVM** | **GLM** | **PAM** | **RF** | **LDA** | **DL** |
| --- | --- | --- | --- | --- | --- | --- |
| **AUC**  **95% CI** | 0.9955  (0.8000-1) | 0.9785  (0.8000-1) | 0.9839  (0.8000-1) | 0.9643  (0.8000-1) | 0.8422  (0.6422-1) | 0.9965  (0.8000-1) |
| **Sensitivity** | 0.9500 | 0.9200 | 0.9000 | 0.8750 | 0.9200 | 0.9800 |
| **Specificity** | 0.9200 | 0.9000 | 0.9500 | 0.9750 | 0.9000 | 0.9800 |

Support Vector Machine (SVM), Generalized Linear Model (GLM), Prediction Analysis for Microarrays (PAM), Random Forest (RF), Linear Discriminant Analysis (LDA) and Deep Learning (DL)

Important predictors in order:

**SVM:** cg05800065, cg04515524, cg26856451, cg12949483, cg00106073

**GLM:** cg00106073, cg26856451, cg00853940, cg05800065, cg16251399

**PAM:** cg26856451, cg19819404, cg04515524, cg27119318, cg12949483

**RF:** cg26856451, cg08829299, cg04515524, cg02356786, cg17160660

**LDA:** cg26856451, cg04515524, cg05800065, cg12949483, cg27119318

**DL:** cg00106073, cg04515524, cg20008763, cg01819759, cg00853940

*This threshold value based on recommendation for genome-wide testing i.e. (FDR p-value <5X10^-8^
