# Supplementary material for: Artificial intelligence and leukocyte epigenomics: Evaluation and prediction of late-onset Alzheimer’s disease
Source: PLoS One. 2021 Mar 31;16(3):e0248375. doi: 10.1371/journal.pone.0248375 (PMC8011726; doi:10.1371/journal.pone.0248375)
Supplement: S6 Table — (DOCX) [file pone.0248375.s006.docx]

**Supplemental Table S6**: Alzheimer’s disease prediction based on Intergenic/extagenic CpG markers (stringent* significance threshold)

| **Parameter** | **SVM** | **GLM** | **PAM** | **RF** | **LDA** | **DL** |
| --- | --- | --- | --- | --- | --- | --- |
| **AUC**  **95% CI** | 0.9925  (0.8000-1) | 0.9552  (0.8000-1) | 0.9708  (0.8000-1) | 0.9740  (0.8000-1) | 0.9677  (0.8000-1) | 0.9865  (0.8000-1) |
| **Sensitivity** | 0.9600 | 0.9300 | 0.9100 | 0.9000 | 0.9200 | 0.9800 |
| **Specificity** | 0.9300 | 0.9200 | 0.9500 | 0.9750 | 0.9000 | 0.9800 |

Support Vector Machine (SVM), Generalized Linear Model (GLM), Prediction Analysis for Microarrays (PAM), Random Forest (RF), Linear Discriminant Analysis (LDA) and Deep Learning (DL)

Important predictors in order:

**SVM:** cg04299067, cg02147364, cg27128435, cg07996016, cg03330558

**GLM:** cg20961783, cg13398715, cg06745364, cg14367592, cg10281502

**PAM:** cg02147364, cg15711973, cg20961783, cg04299067, cg06745364

**RF:** cg03330558, cg20961783, cg06745364, cg24184022, cg04299067

**LDA:** cg02147364, cg04299067, cg17125837, cg27055313, cg10281502

**DL:** cg06745364, cg20961783, cg02147364, cg13398715, cg00786635

*Each CpG marker used in this analysis was differentially methylated in AD compared to controls using stringent significance threshold for genome-wide study i.e. FDR p-value <5X10^-8^.
