# Supplementary material for: Artificial intelligence and leukocyte epigenomics: Evaluation and prediction of late-onset Alzheimer’s disease
Source: PLoS One. 2021 Mar 31;16(3):e0248375. doi: 10.1371/journal.pone.0248375 (PMC8011726; doi:10.1371/journal.pone.0248375)
Supplement: S7 Table — (DOCX) [file pone.0248375.s007.docx]

**Supplemental Table S7:** Differentially methylated genes enriched under molecular pathways in Alzheimer’s disease (Ingenuity pathway analysis).

| **Molecular pathways** | **Genes** | **p-value** | **Reference** |
| --- | --- | --- | --- |
| Cardiac Hypertrophy Signaling (Enhanced) | *ADRA2B, FGF18, FGF22, MYC, WNT9B* | 6.25E-3 | ^1^ |
| Sirtuin Signaling Pathway | *CPS1, MYC, SIRT4, TIMM13* | 2.10E-2 | ^2^ |
| FGF Signaling | *CREB5, FGF18, FGF22, PTPN6* | 4.13E-3 | ^3^ |
| Wnt/β-catenin Signaling | *MYC, SOX14, WNT9B* | 3.14E-2 | ^4^ |
| Neuregulin Signaling | *MYC, NRG1* | 2.00E-2 | ^5^ |

**References**

1. Perales-Puchalt J, Vidoni ML, Llibre Rodriguez J, Vidoni ED, Billinger S, Burns J *et al.* Cardiovascular health and dementia incidence among older adults in Latin America: results from the 10/66 Study. *Int J Geriatr Psychiatry* 2019.

2. Bonda DJ, Lee HG, Camins A, Pallas M, Casadesus G, Smith MA *et al.* The sirtuin pathway in ageing and Alzheimer disease: mechanistic and therapeutic considerations. *Lancet Neurol* 2011; **10**(3)**:** 275-279.

3. Li JS, Yao ZX. Modulation of FGF receptor signaling as an intervention and potential therapy for myelin breakdown in Alzheimer's disease. *Med Hypotheses* 2013; **80**(4)**:** 341-344.

4. Vallee A, Lecarpentier Y. Alzheimer Disease: Crosstalk between the Canonical Wnt/Beta-Catenin Pathway and PPARs Alpha and Gamma. *Front Neurosci* 2016; **10:** 459.

5. Jiang Q, Chen S, Hu C, Huang P, Shen H, Zhao W. Neuregulin-1 (Nrg1) signaling has a preventive role and is altered in the frontal cortex under the pathological conditions of Alzheimer's disease. *Mol Med Rep* 2016; **14**(3)**:** 2614-2624.
