# Supplementary material for: Artificial intelligence and leukocyte epigenomics: Evaluation and prediction of late-onset Alzheimer’s disease
Source: PLoS One. 2021 Mar 31;16(3):e0248375. doi: 10.1371/journal.pone.0248375 (PMC8011726; doi:10.1371/journal.pone.0248375)
Supplement: S8 Table — (DOCX) [file pone.0248375.s008.docx]

**Supplemental Table S8:** Differentially methylated genes enriched in Disease pathways of Alzheimer’s disease (Ingenuity pathway analysis)

| **Disease pathway** | **Genes** | **p-value** | **Reference** |
| --- | --- | --- | --- |
| Abnormal morphology of cerebral cortex | *CR1L, CTSV, APAF1, SS18L1* | 8.90E-4 | ^1^ |
| Gliosis | *S1PR1, CTSV, MYC, SCYL3* | 3.42E-4 | ^2^ |
| Hydrocephalus | *KIF19, ADRA2B, CYP1B1, APAF1, MYB* | 5.61E-4 | ^3^ |
| Morphology of nervous system | *SCYL3, FGF22, PTPRC, S1PR1, CR1L, NRG1, CTSV, DISC1, APAF1, SS18L1, KIF26A, CDK20, MYB, MYC, LMNA, HOXB8* | 3.98E-3 | ^4^ |
| Ventricular hypertrophy and dilated cardiomyopathy | *CTSV, PRMT5, IRX4, LMNA, MYC, TRIM54* | 8.34E-2 | ^5^ |
| Inflammatory response | *LTB4R, S1PR1, PTPN6, VAV1, MYB, MYC* | 8.33E-4 | ^6^ |

**References**

1. Bakkour A, Morris JC, Wolk DA, Dickerson BC. The effects of aging and Alzheimer's disease on cerebral cortical anatomy: specificity and differential relationships with cognition. *Neuroimage* 2013; **76:** 332-344.

2. Leyns CEG, Holtzman DM. Glial contributions to neurodegeneration in tauopathies. *Mol Neurodegener* 2017; **12**(1)**:** 50.

3. Azuma S, Kazui H, Kanemoto H, Suzuki Y, Sato S, Suehiro T *et al.* Cerebral blood flow and Alzheimer's disease-related biomarkers in cerebrospinal fluid in idiopathic normal pressure hydrocephalus. *Psychogeriatrics* 2019.

4. Ohnishi T, Matsuda H, Tabira T, Asada T, Uno M. Changes in brain morphology in Alzheimer disease and normal aging: is Alzheimer disease an exaggerated aging process? *AJNR Am J Neuroradiol* 2001; **22**(9)**:** 1680-1685.

5. Troncone L, Luciani M, Coggins M, Wilker EH, Ho CY, Codispoti KE *et al.* Abeta Amyloid Pathology Affects the Hearts of Patients With Alzheimer's Disease: Mind the Heart. *J Am Coll Cardiol* 2016; **68**(22)**:** 2395-2407.

6. Newcombe EA, Camats-Perna J, Silva ML, Valmas N, Huat TJ, Medeiros R. Inflammation: the link between comorbidities, genetics, and Alzheimer's disease. *J Neuroinflammation* 2018; **15**(1)**:** 276.
