# Supplementary material for: Artificial intelligence and leukocyte epigenomics: Evaluation and prediction of late-onset Alzheimer’s disease
Source: PLoS One. 2021 Mar 31;16(3):e0248375. doi: 10.1371/journal.pone.0248375 (PMC8011726; doi:10.1371/journal.pone.0248375)
Supplement: S9 Table — (DOCX) [file pone.0248375.s009.docx]

**Supplemental Table S9:** List of few genes that were found to be significantly differentially methylated and references indicate a known or plausible link to AD

| **Gene** | **Role** | **Reference** |
| --- | --- | --- |
| *CR1L* | Associated with late-onset AD | ^1^ |
| *MYC* | Seen in dystrophic neurites and neurons with neurofibrillary tangles in AD | ^2^ |
| *NRG1* | Polymorphisms in NRG1 is associated with increasing genetic risk to psychosis in late onset AD | ^3^ |
| *LMNA* | Polymorphisms in LMNA is associated with AD and cognitive functions | ^4^ ^5^ |
| *ELOVL4* | Mutations cause spinocerebellar ataxia, impaired neural development, neuronal dysfunction, hyper-excitability, seizures | ^6^ |
| *MYB* | Plays role in neuronal apoptosis | ^7^ |
| *AGPAT1* | Polymorphisms in AGPAT1 is associated with AD | ^8^ |
| *NSG1 (NEEP21)* | Plays role in neuronal vesicle trafficking | ^9^ |

**References**

1. Kucukkilic E, Brookes K, Barber I, Guetta-Baranes T, Consortium A, Morgan K *et al.* Complement receptor 1 gene (CR1) intragenic duplication and risk of Alzheimer's disease. *Hum Genet* 2018; **137**(4)**:** 305-314.

2. Ferrer I, Blanco R, Carmona M, Puig B. Phosphorylated c-MYC expression in Alzheimer disease, Pick's disease, progressive supranuclear palsy and corticobasal degeneration. *Neuropathol Appl Neurobiol* 2001; **27**(5)**:** 343-351.

3. Go RC, Perry RT, Wiener H, Bassett SS, Blacker D, Devlin B *et al.* Neuregulin-1 polymorphism in late onset Alzheimer's disease families with psychoses. *Am J Med Genet B Neuropsychiatr Genet* 2005; **139B**(1)**:** 28-32.

4. Schjeide BM, McQueen MB, Mullin K, DiVito J, Hogan MF, Parkinson M *et al.* Assessment of Alzheimer's disease case-control associations using family-based methods. *Neurogenetics* 2009; **10**(1)**:** 19-25.

5. Cluett C, Brayne C, Clarke R, Evans G, Matthews F, Rubinsztein DC *et al.* Polymorphisms in LMNA and near a SERPINA gene cluster are associated with cognitive function in older people. *Neurobiol Aging* 2010; **31**(9)**:** 1563-1568.

6. Bazan NG. Docosanoids and elovanoids from omega-3 fatty acids are pro-homeostatic modulators of inflammatory responses, cell damage and neuroprotection. *Mol Aspects Med* 2018; **64:** 18-33.

7. Liu DX, Biswas SC, Greene LA. B-myb and C-myb play required roles in neuronal apoptosis evoked by nerve growth factor deprivation and DNA damage. *J Neurosci* 2004; **24**(40)**:** 8720-8725.

8. Sherva R, Baldwin CT, Inzelberg R, Vardarajan B, Cupples LA, Lunetta K *et al.* Identification of novel candidate genes for Alzheimer's disease by autozygosity mapping using genome wide SNP data. *J Alzheimers Dis* 2011; **23**(2)**:** 349-359.

9. Muthusamy N, Chen YJ, Yin DM, Mei L, Bergson C. Complementary roles of the neuron-enriched endosomal proteins NEEP21 and calcyon in neuronal vesicle trafficking. *J Neurochem* 2015; **132**(1)**:** 20-31.
