# Supplementary material for: Artificial intelligence and leukocyte epigenomics: Evaluation and prediction of late-onset Alzheimer’s disease
Source: PLoS One. 2021 Mar 31;16(3):e0248375. doi: 10.1371/journal.pone.0248375 (PMC8011726; doi:10.1371/journal.pone.0248375)
Supplement: S1 File — (DOCX) [file pone.0248375.s010.docx]

**Supplementary Methods**

**Genome-wide methylation scan using the Infinium MethylationEPIC array BeadChips.** Control and case samples were randomized on the arrays to minimize batch effects. MethylationEPIC array processing and methylome profiling were performed according to the manufacturer's protocol. Fluorescently-stained BeadChips were imaged using the Illumina iScan. Data was analyzed with GenomeStudio Software (Illumina) for methylation analysis. Data preprocessing and quality control were performed including examination of the background signal intensity of negative controls, the methylated and unmethylated signals, and the ratio of the methylated and unmethylated signal intensities. The β-value, an estimate of the methylation level at each CpG locus, was calculated as follows: the intensity of the methylated cytosine allele divided by the sum of the intensity of the methylated plus unmethylated alleles.

**Statistical and Bioinformatic analysis.** To identify the most differentially methylated cytosines a False Discovery Rate (FDR) p-value (also called ‘q’-value) threshold <0.05 with a Benjamini-Hochberg correction for multiple testing was utilized. Further, a >1.5-fold threshold change in methylation was added to increase stringency and to identify methylation changes that were more likely to be biologically significant. The area under the receiver operating characteristics curve (AUC) and 95% CI was calculated for each individual CpG loci to determine its diagnostic performance for AD detection. We first evaluated the predictive accuracy of intragenic CpG markers only and then separately assessed the performance of intergenic sites not currently known to be linked to any genes.

**Removal of confounding factors.** To avoid potential confounding factors, probes associated with X and Y chromosomes and/or containing SNPs in the probe sequence (dbSNP entries near or within the probe sequence, i.e., within 10 bp of the CpG site) were excluded from further analysis^1-3^. Probes targeting CpG loci associated with SNPs near or within the probe sequence may influence corresponding methylated probes ^4^. The remaining CpG sites were then analyzed. Data were normalized using the Controls Normalization Method. To minimize batch effects, all samples were processed together.

**Principal component analysis** **(PCA).** PCA was used for dimensional reduction. Partial least squares discriminant analysis (PLS-DA) was derived from rotating the principal components in the PCA analysis to identify the optimal combination of components for discriminating the study and control groups ^5^. We used these approaches to determine whether epigenetic markers significantly differentiated AD from control groups^6^. Permutation testing was performed to determine whether the observed separation of the AD and control groups in the PLS-DA analysis was statistically significant.

**Logistic Regression Analysis.** Conventional logistic regression analysis for AD prediction based on CpG methylation was also performed using the MetaboAnalyst v4.0 ^6^. Cross validation analysis was done so that screening performance in a subsequent validation group could be determined.

Sum-normalized, log-transformed, and auto-scaled epigenetics data were further subjected to logistic regression analysis using the Biomarker function in MetaboAnalyst v4.0. To select the optimal predictor variables to be used in the logistic regression, Least Absolute Shrinkage and Selection Operator (LASSO) and stepwise variable selection were utilized for optimizing all the model components. The logistic regression models based on CpG loci subsets were developed with 10-fold cross-validation. The area under the receiver operating characteristics curve (AUC), sensitivity and specificity values were calculated for the assessment of model performance**.**

**Gene ontology analysis and functional enrichment.** Genes with differentially methylated (FDR p-value ≤0.05) CpG sites were analyzed using the Ingenuity Pathway Analysis (IPA) software (Qiagen) to identify biological functions or interacting molecular networks. All CpGs without mapping IDs in IPA were excluded from the IPA analysis. Only genes for which Entrez identifiers are available were further analyzed. Over-represented canonical pathways, biological processes, and molecular processes were determined.

**AD prediction based on Artificial Intelligence approaches.** Combinations of individual CpG markers that were significantly (Benjamini- Hochberg FDR p-value < 0.05) epigenetically modified in AD were used to calculate AUC (95% CI), sensitivity, and specificity with AI analysis. Further, AI prediction was repeated but only CpG loci that met stringently defined thresholds for methylation differences between AD and controls (i.e. p-value <5x10^-8^) were included. This threshold has been recommended to ensure the generalizability of results with GWAS studies^7^. Analyses were repeated using this threshold for each of the six ML approaches.

**Data Preparation**

Missing values were identified and replaced with a value set at half of the minimum positive value in the original data. This assumption was made because most missing values are due to a low level of methylation. Further, the log value of each CpG β-value was centered by its mean and auto-scaled by its standard deviation. The quantile normalization method was used to reduce sample-to-sample variation.

**Artificial Intelligence Algorithms**

**Deep Learning (DL):** Deep-learning methods are representation-learning (or feature-learning) methods with multiple levels of representation, obtained by composing simple but non-linear modules that each transform the representation at one level (starting with the raw input) into a representation at a higher, slightly more abstract level. Using enough such transformations, very complex functions can be learned. For classification tasks, higher layers of representation prioritize aspects of the input that are important for discrimination while irrelevant variations are suppressed. This hierarchical learning process is very powerful as it allows a system to comprehend and learn complex representations directly from the raw data ^8^, thus making the approach useful in many disciplines ^9^.

Similar to other feed-forward artificial neural networks (ANNs), DL employs more than one hidden layer (y) that connects the input (x) and output layers (z) via a weight (W) matrix. The activation value of the hidden layer (y) can be calculated by sigmoid of the multiplication of the input sample x with the weight matrix W and bias b. The transpose of the weight matrix W and the bias b can then be used to construct the output (z) layer. The optimal set of the weight matrix ‘W’ and bias ‘b’ is expected to minimize the difference between the input layer (x) and the output layer (z).

**Random Forest (RF):** RF is a supervised classification algorithm. There is a direct relationship between the number of trees in the forest and the precision: the larger the number of trees, the more accurate the results ^10^. The difference between the Random Forest algorithm and the decision tree algorithm is that with Random Forest, the processes of finding the root node and splitting the feature nodes occur randomly. The decision tree is a decision support tool that uses a tree-like graph to show the possible consequences. If one enters a training dataset with targets and features into the decision tree, it will formulate a set of rules. Overfitting is one critical problem that may decrease accuracy in decision trees. For Random Forest algorithms, however, if there are enough trees in the forest, the classifier will not overfit model ^10^. Another advantage is the classifier in Random Forest can handle missing values, and the last advantage is that the Random Forest classifier can be modeled for categorical values ^10^.

**Support vector machine (SVM):** SVMs ^11^ is a relatively new type of learning algorithm. They display remarkably robust performance concerning sparse and noisy data making SVM the system of choice in several applications from text categorization to the field of bioinformatics. When used for classification, they separate a given set of binary labeled training data with a hyper-plane that is maximally distant from the two data groups (known as ‘the maximal margin hyper-plane) ^12^. For cases in which no linear separation is possible, they can work in combination with the technique of ‘kernels’ that automatically realizes a non-linear mapping to a feature space. The hyper-plane found by the SVM in feature space corresponds to a non-linear decision boundary in the input space.

**Linear Discriminant Analysis (LDA):** Principal Component Analysis (PCA) and Linear Discriminant Analysis (LDA) are two commonly used techniques for data classification and dimensional reduction. Linear Discriminant Analysis comfortably handles situations in which the within-class frequencies are unequal. This method maximizes the ratio of between-class variance to the within-class variance in any particular data set and is thereby able to guarantee maximal separability ^13^.

**Prediction Analysis for Microarrays (PAM):** is a statistical technique for class prediction using gene expression data using nearest shrunken centroids. This method identifies the subsets of genes that best characterize each class.

**Generalized Linear Model (GLM):** The generalized linear models (GLMs) are a broad class of models that include linear regression, ANOVA, Poisson regression, log-linear models, etc. It is a way of unifying these different statistical approaches. The response (outcome) variables do not have to be normally distributed. There are some limitations of GLMs, such as, linear function, which can have only a linear predictor in the systematic component, and responses must be independent.

**Software Tools**

We used h2o R package ^14^ (<https://cran.r-project.org/web/packages/h2o/h2o.pdf>, Author The H2O.ai team Maintainer Tom Kraljevic <tomk@0xdata.com>) to tune the parameters of the DL model and the caret R package (<https://cran.r-project.org/web/packages/caret/caret.pdf>, Maintainer Max Kuhn <mxkuhn@gmail.com> , December 10, 2017 ) to tune the parameters in the other artificial intelligence models ^15^.

The variable importance functions *varimp* in h2o and *varImp* in caret R packages were utilized to rank the model’s features in each of the predictive algorithms.

We used pROC R package to compute area under the curve (AUC) of a receiver-operating characteristic (ROC) curve, specificity and sensitivity to assess the overall performance of the models ^16^.

**Modeling & Evaluation**

The data were split into training and testing sets to train the model first with a portion of the data and then validate the results by using the remaining portion of the data that was set aside. The data split was 80% for the training and 20% for the testing groups is generally used in medium-sized data sets. 10-fold cross-validation on the 80% training data was performed during the model construction process. Besides, the process of dichotomizing into test and validation groups was repeated ten times, and the average AUC, sensitivity, specificity, and 95% confidence intervals for the test set were calculated.

Several parameters were used to tune the models: Number of trees for RF, classification cost for SVM, the threshold amount for shrinking toward the centroid for PAM, and for the DL model: a) Epochs (number of passes of the full training set), b) l1 (penalty to converge the weights of the model to 0), c) l2 (penalty to prevent the enlargement of the weights), d) input dropout ratio (ratio of ignored neurons in the input layer during training), e) number of hidden layers. In addition to, l1 and l2 parameters, *input_dropout_ratio* was used as the third parameter to avoid overfitting in the DL model which controls the amount of input layer neurons that are randomly dropped (set to zero). This process minimizes overfitting concerning the input data (useful for high-dimensional noisy data). The key point was to randomly drop units (along with their connections) from the neural network during training ^17^. This prevents units from excessive co-adaptation. With the help of these three approaches, we avoided the most significant complication DL analysis i.e. overfitting ^17^.

**Ranking Important Features**

The contribution of a feature (predictor) to the model performance was considered using a model-based approach. We ranked the importance of the features in each of the predictive algorithms by using the variable importance functions *varimp* in h2o and *varImp* in caret R computer packages.

**The codes used for the analysis is provided below.**

**1-SVM**

###############################load the dataset

prostate_df <- read.csv(file="Alzheimer1.csv",check.names = T, stringsAsFactors = TRUE)

prostate_df <- prostate_df[,-1]# reomve sampel labels

prostate_df$subtype <- as.factor(ifelse(prostate_df$subtype==1,1,0))

#prostate_df[,ncol(prostate_df) -1] <- factor(prostate_df[,ncol(prostate_df) -1])

#prostate_df[,ncol(prostate_df) -2] <- factor(prostate_df[,ncol(prostate_df) -2])

#prostate_df[,ncol(prostate_df) -3] <- factor(prostate_df[,ncol(prostate_df) -3])

#prostate_df[,ncol(prostate_df) -4] <- factor(prostate_df[,ncol(prostate_df) -4])

#prostate_df[c(ncol(prostate_df) -1)] <- factor(prostate_df[c(ncol(prostate_df) -1)])

#prostate_df[c(ncol(prostate_df) -2)] <- factor(prostate_df[c(ncol(prostate_df) -2)])

#prostate_df[c(ncol(prostate_df) -3)] <- factor(prostate_df[c(ncol(prostate_df) -3)])

#prostate_df[c(ncol(prostate_df) -4)] <- factor(prostate_df[c(ncol(prostate_df) -4)])

##################

# Quantile normalization, each column corresponds to a sample and each row is a metabolites.

#metadatanorm=normalize.quantiles(t(as.matrix(prostate_df[,-ncol(prostate_df)])))

#[,-ncol(prostate_df)]

#################

# calculate the pre-process parameters from the dataset

preprocessParams <- preProcess(prostate_df[,1:ncol(prostate_df) -1], method=c("center", "scale"))

# summarize transform parameters

print(preprocessParams)

# transform the dataset using the parameters

transformed <- predict(preprocessParams, prostate_df[,1:ncol(prostate_df) -1])

df <- as.data.frame (metadatanorm)

df2 <- cbind (df, prostate_df$subtype)

names(df2) <- names(prostate_df)

prostate_df <- df2

response <- 'subtype'

predictors <- setdiff(names(prostate_df), response)

performance_training=matrix( rep( 0, len=3), nrow = 3) #AUC SENS SPECF

performance_testing=matrix( rep( 0, len=8), nrow = 8) # ROC SENS SPEC

performance=matrix(rep( 0, len=8), nrow = 8) # ROC SENS SPEC

performance_testing_list=list()

performance_training_list=list()

for (k in 1:10) {

###############Shuffle stat first

rand <- sample(nrow(prostate_df))

prostate_df=prostate_df[rand, ]

###############Randomly Split the data in to training and testing

trainIndex <- createDataPartition(prostate_df$subtype, p = .8,list = FALSE,times = 1)

irisTrain <- prostate_df[ trainIndex,]

irisTest <- prostate_df[-trainIndex,]

irisTrain$subtype=as.factor(paste0("X",irisTrain$subtype))

irisTest$subtype=as.factor(paste0("X",irisTest$subtype))

################################Training and tunning parameters

# prepare training scheme

control <- trainControl(method="CV", number=10,classProbs = TRUE,summaryFunction =

twoClassSummary)

#3- SVM ALGORITHM

set.seed(9)

fit.svm <- train(subtype~., data=irisTrain, method="svmRadial", trControl=control,metric="ROC")

#assign(paste0("fit.svm",k),train(subtype~., data=irisTrain, method="svmRadical", trControl=control,metric="ROC"))

performance_training[1,1]=max(fit.svm$results$ROC) #AUC

performance_training[2,1]=fit.svm$results$Sens[which.max(fit.svm$results$ROC)]# sen

performance_training[3,1]=fit.svm$results$Spec[which.max(fit.svm$results$ROC)]# spec

importance <- varImp(fit.svm, scale=FALSE)

# summarize importance

print(importance)

# plot importance

plot(importance)

#Model Testing

svmClasses <- predict( fit.svm, newdata = irisTest,type="prob")

svmClasses1 <- predict( fit.svm, newdata = irisTest)

svmConfusion=confusionMatrix(data = svmClasses1, irisTest$subtype)

svm.ROC <-

roc(predictor=svmClasses$X1,response=irisTest$subtype,ci=TRUE,levels=rev(levels(irisTest$subtype)))

print(svm.ROC$ci)

performance_testing[1,1]=as.numeric(svm.ROC$auc)#AUC

performance_testing[2,1]=svmConfusion$byClass[1]#SENS

performance_testing[3,1]=svmConfusion$byClass[2]#SPEC

performance_testing[4,1]=svmConfusion$overall[1]#accuracy

performance_testing[5,1]=svmConfusion$byClass[5]#precision

performance_testing[6,1]=svmConfusion$byClass[6]#recall = sens

performance_testing[7,1]=svmConfusion$byClass[7]#F1

performance_testing[8,1]=svmConfusion$byClass[11]#BALANCED ACCURACY

performance_testing_list[[k]]=performance_testing

performance_training_list[[k]]=performance_training

performance_training=matrix( rep( 0, len=3), nrow = 3) #AUC SENS SPECF

performance_testing=matrix( rep( 0, len=8), nrow = 8) # ROC SENS SPEC

}

######################AUC plot_testing

list_test=performance_testing_list

list_train=performance_training_list

ee=lapply(list_test, function(x) x[1,])

output <- do.call(rbind,lapply(ee,matrix,ncol=1,byrow=TRUE))

AUC_mean=apply(output,2,mean)

AUC_mean=data.frame(value=AUC_mean,Algorithm=c('SVM'))

#colnames(AUC_mean)=c('SVM')

pdf("AUC_mean_testing.pdf")

p<-ggplot(data=AUC_mean, aes(x=Algorithm, y=value)) + geom_bar(stat="identity")

plot(p)

dev.off()

**2-RF**

###############################load the dataset

prostate_df <- read.csv(file="AD-Blood-LOAD.csv",check.names = T, stringsAsFactors = TRUE)

prostate_df <- prostate_df[,-1]# reomve sampel labels

prostate_df$subtype <- as.factor(ifelse(prostate_df$subtype==1,1,0))

##################

# Quantile normalization, each column corresponds to a sample and each row is a metabolites.

#metadatanorm=normalize.quantiles(t(as.matrix(prostate_df[,-ncol(prostate_df)])))

#[,-ncol(prostate_df)]

#################

# calculate the pre-process parameters from the dataset

preprocessParams <- preProcess(prostate_df[,1:ncol(prostate_df) -1], method=c("center", "scale"))

# summarize transform parameters

print(preprocessParams)

# transform the dataset using the parameters

transformed <- predict(preprocessParams, prostate_df[,1:ncol(prostate_df) -1])

df <- as.data.frame (metadatanorm)

df2 <- cbind (df, prostate_df$subtype)

names(df2) <- names(prostate_df)

prostate_df <- df2

response <- 'subtype'

predictors <- setdiff(names(prostate_df), response)

performance_training=matrix( rep( 0, len=3), nrow = 3) #AUC SENS SPECF

performance_testing=matrix( rep( 0, len=8), nrow = 8) # ROC SENS SPEC

performance=matrix(rep( 0, len=8), nrow = 8) # ROC SENS SPEC

performance_testing_list=list()

performance_training_list=list()

for (k in 1:10) {

###############Shuffle stat first

rand <- sample(nrow(prostate_df))

prostate_df=prostate_df[rand, ]

###############Randomly Split the data in to training and testing

trainIndex <- createDataPartition(prostate_df$subtype, p = .8,list = FALSE,times = 1)

irisTrain <- prostate_df[ trainIndex,]

irisTest <- prostate_df[-trainIndex,]

irisTrain$subtype=as.factor(paste0("X",irisTrain$subtype))

irisTest$subtype=as.factor(paste0("X",irisTest$subtype))

################################Training and tunning parameters

# prepare training scheme

control <- trainControl(method="cv", number=10,classProbs = TRUE,summaryFunction =

twoClassSummary)

#4-RF ALGORITHM

set.seed(10)

fit.rf <- train(subtype~., data=irisTrain, method="rf", trControl=control,metric="ROC")

performance_training[1,1]=max(fit.rf$results$ROC) #AUC

performance_training[2,1]=fit.rf$results$Sens[which.max(fit.rf$results$ROC)]# sen

performance_training[3,1]=fit.rf$results$Spec[which.max(fit.rf$results$ROC)]# spec

importance <- varImp(fit.rf, scale=FALSE)

# summarize importance

print(importance)

# plot importance

plot(importance)

#Model Testing

rfClasses <- predict( fit.rf, newdata = irisTest,type="prob")

rfClasses1 <- predict( fit.rf, newdata = irisTest)

rfConfusion=confusionMatrix(data = rfClasses1, irisTest$subtype)

rf.ROC <- roc(predictor=rfClasses$X1,response=irisTest$subtype,levels=rev(levels(irisTest$subtype)))

performance_testing[1,1]=as.numeric(rf.ROC$auc)#AUC

performance_testing[2,1]=rfConfusion$byClass[1]#SENS

performance_testing[3,1]=rfConfusion$byClass[2]#SPEC

performance_testing[4,1]=rfConfusion$overall[1]#accuracy

performance_testing[5,1]=rfConfusion$byClass[5]#precision

performance_testing[6,1]=rfConfusion$byClass[6]#recall = sens

performance_testing[7,1]=rfConfusion$byClass[7]#F1

performance_testing[8,1]=rfConfusion$byClass[11]#BALANCED ACCURACY

performance_testing_list[[k]]=performance_testing

performance_training_list[[k]]=performance_training

performance_training=matrix( rep( 0, len=3), nrow = 3) #AUC SENS SPECF

performance_testing=matrix( rep( 0, len=8), nrow = 8) # ROC SENS SPEC

}

######################AUC plot_testing

list_test=performance_testing_list

list_train=performance_training_list

ee=lapply(list_test, function(x) x[1,])

output <- do.call(rbind,lapply(ee,matrix,ncol=1,byrow=TRUE))

AUC_mean=apply(output,2,mean)

AUC_mean=data.frame(value=AUC_mean,Algorithm=c('RF'))

#colnames(AUC_mean)=c('SVM')

pdf("AUC_mean_testing.pdf")

p<-ggplot(data=AUC_mean, aes(x=Algorithm, y=value)) + geom_bar(stat="identity")

plot(p)

dev.off()

**3-PAM**

###############################load the dataset

prostate_df <- read.csv(file="AD-Blood-LOAD.csv",check.names = T, stringsAsFactors = TRUE)

prostate_df <- prostate_df[,-1]# reomve sampel labels

prostate_df$subtype <- as.factor(ifelse(prostate_df$subtype==1,1,0))

##################

# Quantile normalization, each column corresponds to a sample and each row is a metabolites.

#metadatanorm=normalize.quantiles(t(as.matrix(prostate_df[,-ncol(prostate_df)])))

#[,-ncol(prostate_df)]

#################

# calculate the pre-process parameters from the dataset

preprocessParams <- preProcess(prostate_df[,1:ncol(prostate_df) -1], method=c("center", "scale"))

# summarize transform parameters

print(preprocessParams)

# transform the dataset using the parameters

transformed <- predict(preprocessParams, prostate_df[,1:ncol(prostate_df) -1])

#prostate_df[1:ncol(prostate_df) -1]=t(metadatanorm)

#prostate_df[,-ncol(prostate_df)]

metadatanorm=normalize.quantiles(data.matrix(transformed))

df <- as.data.frame (metadatanorm)

df2 <- cbind (df, prostate_df$subtype)

names(df2) <- names(prostate_df)

prostate_df <- df2

response <- 'subtype'

predictors <- setdiff(names(prostate_df), response)

performance_training=matrix( rep( 0, len=3), nrow = 3) #AUC SENS SPECF

performance_testing=matrix( rep( 0, len=8), nrow = 8) # ROC SENS SPEC

performance=matrix(rep( 0, len=8), nrow = 8) # ROC SENS SPEC

performance_testing_list=list()

performance_training_list=list()

for (k in 1:10) {

###############Shuffle stat first

rand <- sample(nrow(prostate_df))

prostate_df=prostate_df[rand, ]

###############Randomly Split the data in to training and testing

trainIndex <- createDataPartition(prostate_df$subtype, p = .8,list = FALSE,times = 1)

irisTrain <- prostate_df[ trainIndex,]

irisTest <- prostate_df[-trainIndex,]

irisTrain$subtype=as.factor(paste0("X",irisTrain$subtype))

irisTest$subtype=as.factor(paste0("X",irisTest$subtype))

################################Training and tunning parameters

# prepare training scheme

control <- trainControl(method="cv", number=10,classProbs = TRUE,summaryFunction =

twoClassSummary)

#6- PAM ALGORITHM

set.seed(10)

fit.pam <- train(subtype~., data=irisTrain, method="pam", trControl=control,metric="ROC")#plr

performance_training[1,1]=max(fit.pam$results$ROC) #AUC

performance_training[2,1]=fit.pam$results$Sens[which.max(fit.pam$results$ROC)]# sen

performance_training[3,1]=fit.pam$results$Spec[which.max(fit.pam$results$ROC)]# spec

importance <- varImp(fit.svm, scale=FALSE)

# summarize importance

print(importance)

# plot importance

plot(importance)

#Model Testing

pamClasses <- predict( fit.pam, newdata = irisTest,type="prob")

pamClasses1 <- predict( fit.pam, newdata = irisTest)

pamConfusion=confusionMatrix(data = pamClasses1, irisTest$subtype)

pam.ROC <-

roc(predictor=pamClasses$X1,response=irisTest$subtype,levels=rev(levels(irisTest$subtype)))

performance_testing[1,1]=as.numeric(pam.ROC$auc)#AUC

performance_testing[2,1]=pamConfusion$byClass[1]#SENS

performance_testing[3,1]=pamConfusion$byClass[2]#SPEC

performance_testing[4,1]=pamConfusion$overall[1]#accuracy

performance_testing[5,1]=pamConfusion$byClass[5]#precision

performance_testing[6,1]=pamConfusion$byClass[6]#recall = sens

performance_testing[7,1]=pamConfusion$byClass[7]#F1

performance_testing[8,1]=pamConfusion$byClass[11]#BALANCED ACCURACY

performance_testing_list[[k]]=performance_testing

performance_training_list[[k]]=performance_training

performance_training=matrix( rep( 0, len=3), nrow = 3) #AUC SENS SPECF

performance_testing=matrix( rep( 0, len=8), nrow = 8) # ROC SENS SPEC

}

######################AUC plot_testing

list_test=performance_testing_list

list_train=performance_training_list

ee=lapply(list_test, function(x) x[1,])

output <- do.call(rbind,lapply(ee,matrix,ncol=1,byrow=TRUE))

AUC_mean=apply(output,2,mean)

AUC_mean=data.frame(value=AUC_mean,Algorithm=c('PAM'))

#colnames(AUC_mean)=c('SVM')

pdf("AUC_mean_testing.pdf")

p<-ggplot(data=AUC_mean, aes(x=Algorithm, y=value)) + geom_bar(stat="identity")

plot(p)

dev.off()

**4-GLM**

###############################load the dataset

prostate_df <- read.csv(file="AD-Blood-LOAD.csv",check.names = T, stringsAsFactors = TRUE)

prostate_df <- prostate_df[,-1]# reomve sampel labels

prostate_df$subtype <- as.factor(ifelse(prostate_df$subtype==1,1,0))

##################

# Quantile normalization, each column corresponds to a sample and each row is a metabolites.

#metadatanorm=normalize.quantiles(t(as.matrix(prostate_df[,-ncol(prostate_df)])))

#[,-ncol(prostate_df)]

#################

# calculate the pre-process parameters from the dataset

preprocessParams <- preProcess(prostate_df[,1:ncol(prostate_df) -1], method=c("center", "scale"))

# summarize transform parameters

print(preprocessParams)

# transform the dataset using the parameters

transformed <- predict(preprocessParams, prostate_df[,1:ncol(prostate_df) -1])

#prostate_df[1:ncol(prostate_df) -1]=t(metadatanorm)

#prostate_df[,-ncol(prostate_df)]

metadatanorm=normalize.quantiles(data.matrix(transformed))

# for (k in 1:(ncol(prostate_df) -1)) {

# prostate_df[, a] = (data.frame(metadatanorm))[,a]

# }

# prostate_df = metadatanorm[,1:ncol(prostate_df) -1]

df <- as.data.frame (metadatanorm)

df2 <- cbind (df, prostate_df$subtype)

names(df2) <- names(prostate_df)

prostate_df <- df2

response <- 'subtype'

predictors <- setdiff(names(prostate_df), response)

performance_training=matrix( rep( 0, len=3), nrow = 3) #AUC SENS SPECF

performance_testing=matrix( rep( 0, len=8), nrow = 8) # ROC SENS SPEC

performance=matrix(rep( 0, len=8), nrow = 8) # ROC SENS SPEC

performance_testing_list=list()

performance_training_list=list()

for (k in 1:10) {

###############Shuffle stat first

rand <- sample(nrow(prostate_df))

prostate_df=prostate_df[rand, ]

###############Randomly Split the data in to training and testing

trainIndex <- createDataPartition(prostate_df$subtype, p = .8,list = FALSE,times = 1)

irisTrain <- prostate_df[ trainIndex,]

irisTest <- prostate_df[-trainIndex,]

irisTrain$subtype=as.factor(paste0("X",irisTrain$subtype))

irisTest$subtype=as.factor(paste0("X",irisTest$subtype))

################################Training and tunning parameters

# prepare training scheme

control <- trainControl(method="cv", number=10,classProbs = TRUE,summaryFunction =

twoClassSummary)

#2-GLM ALGORITHM

set.seed(10)

#assign(paste0("fit.lda",k),train(subtype~., data=irisTrain, method="pls", trControl=control,metric="ROC"))

fit.glm <- train(subtype~., data=irisTrain, method = 'glmnet', trControl=control,metric="ROC") #loclda

performance_training[1,1]=max(fit.glm$results$ROC)#AUC

performance_training[2,1]=fit.glm$results$Sens[which.max(fit.glm$results$ROC)]# sen

performance_training[3,1]=fit.glm$results$Spec[which.max(fit.glm$results$ROC)]# spec

summary(fit.glm)

importance <- varImp(fit.glm, scale=FALSE)

# summarize importance

print(importance)

# plot importance

plot(importance)

#Model Testing

glmClasses <- predict( fit.glm, newdata = irisTest,type="prob")

glmClasses1 <- predict( fit.glm, newdata = irisTest)

glmConfusion=confusionMatrix(data = glmClasses1, irisTest$subtype)

glm.ROC <- roc(predictor=glmClasses$X1,response=irisTest$subtype,levels=rev(levels(irisTest$subtype)))

performance_testing[1,1]=as.numeric(glm.ROC$auc)#AUC

performance_testing[2,1]=glmConfusion$byClass[1]#SENS

performance_testing[3,1]=glmConfusion$byClass[2]#SPEC

performance_testing[4,1]=glmConfusion$overall[1]#accuracy

performance_testing[5,1]=glmConfusion$byClass[5]#precision

performance_testing[6,1]=glmConfusion$byClass[6]#recall = sens

performance_testing[7,1]=glmConfusion$byClass[7]#F1

performance_testing[8,1]=glmConfusion$byClass[11]#BALANCED ACCURACY

performance_testing_list[[k]]=performance_testing

performance_training_list[[k]]=performance_training

performance_training=matrix( rep( 0, len=3), nrow = 3) #AUC SENS SPECF

performance_testing=matrix( rep( 0, len=8), nrow = 8) # ROC SENS SPEC

}

######################AUC plot_testing

list_test=performance_testing_list

list_train=performance_training_list

ee=lapply(list_test, function(x) x[1,])

output <- do.call(rbind,lapply(ee,matrix,ncol=1,byrow=TRUE))

AUC_mean=apply(output,2,mean)

AUC_mean=data.frame(value=AUC_mean,Algorithm=c('GLM'))

#colnames(AUC_mean)=c('GLM')

pdf("AUC_mean_testing.pdf")

p<-ggplot(data=AUC_mean, aes(x=Algorithm, y=value)) + geom_bar(stat="identity")

plot(p)

dev.off()

**5-LDA**

###############################load the dataset

prostate_df <- read.csv(file="AD-Blood-LOAD.csv",check.names = T, stringsAsFactors = TRUE)

prostate_df <- prostate_df[,-1]# reomve sampel labels

prostate_df$subtype <- as.factor(ifelse(prostate_df$subtype==1,1,0))

##################

# Quantile normalization, each column corresponds to a sample and each row is a metabolites.

#metadatanorm=normalize.quantiles(t(as.matrix(prostate_df[,-ncol(prostate_df)])))

#[,-ncol(prostate_df)]

#################

# calculate the pre-process parameters from the dataset

preprocessParams <- preProcess(prostate_df[,1:ncol(prostate_df) -1], method=c("center", "scale"))

# summarize transform parameters

print(preprocessParams)

# transform the dataset using the parameters

transformed <- predict(preprocessParams, prostate_df[,1:ncol(prostate_df) -1])

#prostate_df[1:ncol(prostate_df) -1]=t(metadatanorm)

#prostate_df[,-ncol(prostate_df)]

metadatanorm=normalize.quantiles(data.matrix(transformed))

# for (k in 1:(ncol(prostate_df) -1)) {

# prostate_df[, a] = (data.frame(metadatanorm))[,a]

# }

# prostate_df = metadatanorm[,1:ncol(prostate_df) -1]

df <- as.data.frame (metadatanorm)

df2 <- cbind (df, prostate_df$subtype)

names(df2) <- names(prostate_df)

prostate_df <- df2

response <- 'subtype'

predictors <- setdiff(names(prostate_df), response)

performance_training=matrix( rep( 0, len=3), nrow = 3) #AUC SENS SPECF

performance_testing=matrix( rep( 0, len=8), nrow = 8) # ROC SENS SPEC

performance=matrix(rep( 0, len=8), nrow = 8) # ROC SENS SPEC

performance_testing_list=list()

performance_training_list=list()

for (k in 1:10) {

###############Shuffle stat first

rand <- sample(nrow(prostate_df))

prostate_df=prostate_df[rand, ]

###############Randomly Split the data in to training and testing

trainIndex <- createDataPartition(prostate_df$subtype, p = .8,list = FALSE,times = 1)

irisTrain <- prostate_df[ trainIndex,]

irisTest <- prostate_df[-trainIndex,]

irisTrain$subtype=as.factor(paste0("X",irisTrain$subtype))

irisTest$subtype=as.factor(paste0("X",irisTest$subtype))

################################Training and tunning parameters

# prepare training scheme

control <- trainControl(method="cv", number=5,classProbs = TRUE,summaryFunction =

twoClassSummary)

#2-LDA ALGORITHM

set.seed(10)

#assign(paste0("fit.lda",k),train(subtype~., data=irisTrain, method="pls", trControl=control,metric="ROC"))

fit.lda <- train(subtype~., data=irisTrain, method = 'lda', trControl=control,metric="ROC") #loclda

performance_training[1,1]=max(fit.lda$results$ROC)#AUC

performance_training[2,1]=fit.lda$results$Sens[which.max(fit.lda$results$ROC)]# sen

performance_training[3,1]=fit.lda$results$Spec[which.max(fit.lda$results$ROC)]# spec

importance <- varImp(fit.lda, scale=FALSE)

# summarize importance

print(importance)

# plot importance

plot(importance)

#Model Testing

ldaClasses <- predict( fit.lda, newdata = irisTest,type="prob")

ldaClasses1 <- predict( fit.lda, newdata = irisTest)

ldaConfusion=confusionMatrix(data = ldaClasses1, irisTest$subtype)

lda.ROC <- roc(predictor=ldaClasses$X1,response=irisTest$subtype,levels=rev(levels(irisTest$subtype)))

performance_testing[1,1]=as.numeric(lda.ROC$auc)#AUC

performance_testing[2,1]=ldaConfusion$byClass[1]#SENS

performance_testing[3,1]=ldaConfusion$byClass[2]#SPEC

performance_testing[4,1]=ldaConfusion$overall[1]#accuracy

performance_testing[5,1]=ldaConfusion$byClass[5]#precision

performance_testing[6,1]=ldaConfusion$byClass[6]#recall = sens

performance_testing[7,1]=ldaConfusion$byClass[7]#F1

performance_testing[8,1]=ldaConfusion$byClass[11]#BALANCED ACCURACY

performance_testing_list[[k]]=performance_testing

performance_training_list[[k]]=performance_training

performance_training=matrix( rep( 0, len=3), nrow = 3) #AUC SENS SPECF

performance_testing=matrix( rep( 0, len=8), nrow = 8) # ROC SENS SPEC

}

######################AUC plot_testing

list_test=performance_testing_list

list_train=performance_training_list

ee=lapply(list_test, function(x) x[1,])

output <- do.call(rbind,lapply(ee,matrix,ncol=1,byrow=TRUE))

AUC_mean=apply(output,2,mean)

AUC_mean=data.frame(value=AUC_mean,Algorithm=c('LDA'))

#colnames(AUC_mean)=c('LDA')

pdf("AUC_mean_testing.pdf")

p<-ggplot(data=AUC_mean, aes(x=Algorithm, y=value)) + geom_bar(stat="identity")

plot(p)

dev.off()

**6-DL**

localH2O = h2o.init(nthreads=-1, max_mem_size="4g")

#h2o.init(nthreads=-1, max_mem_size="4g")

###############################load the dataset

prostate_df <- read.csv(file="AD-Blood-LOAD.csv",check.names = T, stringsAsFactors = TRUE)

prostate_df <- prostate_df[,-1]# reomve sampel labels

prostate_df$subtype <- as.factor(ifelse(prostate_df$subtype==1,1,0))

##################

# Quantile normalization, each column corresponds to a sample and each row is a metabolites.

#metadatanorm=normalize.quantiles(t(as.matrix(prostate_df[,-ncol(prostate_df)])))

#[,-ncol(prostate_df)]

#################

# calculate the pre-process parameters from the dataset

preprocessParams <- preProcess(prostate_df[,1:ncol(prostate_df) -1], method=c("center", "scale"))

# summarize transform parameters

print(preprocessParams)

# transform the dataset using the parameters

transformed <- predict(preprocessParams, prostate_df[,1:ncol(prostate_df) -1])

#prostate_df[1:ncol(prostate_df) -1]=t(metadatanorm)

#prostate_df[,-ncol(prostate_df)]

metadatanorm=normalize.quantiles(data.matrix(transformed))

# for (k in 1:(ncol(prostate_df) -1)) {

# prostate_df[, a] = (data.frame(metadatanorm))[,a]

# }

# prostate_df = metadatanorm[,1:ncol(prostate_df) -1]

df <- as.data.frame (metadatanorm)

df2 <- cbind (df, prostate_df$subtype)

names(df2) <- names(prostate_df)

prostate_df <- df2

response <- 'subtype'

predictors <- setdiff(names(prostate_df), response)

performance_training=matrix( rep( 0, len=3), nrow = 3) #AUC SENS SPECF

performance_testing=matrix( rep( 0, len=8), nrow = 8) # ROC SENS SPEC

performance=matrix(rep( 0, len=8), nrow = 8) # ROC SENS SPEC

performance_testing_list=list()

performance_training_list=list()

for (k in 1:10) {

###############Shuffle stat first

rand <- sample(nrow(prostate_df))

prostate_df=prostate_df[rand, ]

###############Randomly Split the data in to training and testing

trainIndex <- createDataPartition(prostate_df$subtype, p = .8,list = FALSE,times = 1)

irisTrain <- prostate_df[ trainIndex,]

irisTest <- prostate_df[-trainIndex,]

irisTrain$subtype=as.factor(paste0("X",irisTrain$subtype))

irisTest$subtype=as.factor(paste0("X",irisTest$subtype))

################################Training and tunning parameters

# prepare training scheme

control <- trainControl(method="cv", number=10,classProbs = TRUE,summaryFunction =

twoClassSummary)

#7- DL ALGORITHM

prostate.hex<-as.h2o(irisTrain, destination_frame="train.hex")

#valid <- as.h2o(irisTest, destination_frame="test.hex")

#prostate.hex$subtype <- as.factor(prostate.hex$subtype) ##make categorical

#Model Testing

hyper_params <- list(

activation=c("Rectifier","Tanh"),

hidden=list(c(100),c(200),c(10,10),c(20,20),c(50,50),c(30,30,30),c(25,25,25,25)),

input_dropout_ratio=c(0,0.05,0.1),

#hidden_dropout_ratios=c(0.6,0.5,0.6,0.6),

l1=seq(0,1e-4,1e-6),

l2=seq(0,1e-4,1e-6),

train_samples_per_iteration =c(0,-2),

epochs = c(500),

variable_importances=T,

momentum_start=c(0,0.5),

rho=c(0.5,0.99),

quantile_alpha=c(0,1),

huber_alpha=seq(0,1) )

search_criteria = list(strategy = "RandomDiscrete", max_models = 100, stopping_rounds=5,

stopping_tolerance=1e-2)

dl_random_grid <- h2o.grid(

algorithm="deeplearning",

#grid_id = paste("dl_do3",k,sep = "_"),

grid_id = "dl_grid_randome1",

training_frame=prostate.hex,

#validation_frame=valid,

#hidden=c(25,25,25,25),

x=predictors,

y="subtype",

#pretrained_autoencoder="dl_grid_random1_model_42",

#autoencoder = TRUE,

seed=1,

#adaptive_rate=T, ## manually tuned learning rate

#momentum_start=0.5, ## manually tuned momentum

#momentum_stable=0.9,

#momentum_ramp=1e7,

variable_importances=TRUE,

export_weights_and_biases=T,

standardize=T,

stopping_metric="misclassification",

stopping_tolerance=1e-2, ## stop when logloss does not improve by >=1% for 2 scoring events

stopping_rounds=2,

#score_validation_samples=10000, ## downsample validation set for faster scoring

score_duty_cycle=0.025, ## don't score more than 2.5% of the wall time

#max_w2=10, ## can help improve stability for Rectifier

hyper_params = hyper_params,

search_criteria = search_criteria,

nfolds=10

)

grid <- h2o.getGrid("dl_grid_randome1",sort_by="mse",decreasing=FALSE)

grid@summary_table[1,]

best_model <- h2o.getModel(grid@model_ids[[1]]) ## model with lowest logloss

h2o.varimp_plot(best_model, num_of_features = NULL)

performance_training[1,1]=as.numeric(best_model@model$cross_validation_metrics_summary$mean)[2] #AUC

performance_training[2,1]=as.numeric(best_model@model$cross_validation_metrics_summary$mean)[17]# sen

performance_training[3,1]=as.numeric(best_model@model$cross_validation_metrics_summary$mean)[19]# spec

perf=h2o.performance(best_model,as.h2o(irisTest, destination_frame="test.hex"))

performance_testing[1,1]=as.numeric(h2o.auc(perf,h2o.find_threshold_by_max_metric(perf,"f1"))[[1]])

#AUC

performance_testing[2,1]=as.numeric(h2o.sensitivity(perf,h2o.find_threshold_by_max_metric(perf,"f1"))[[1]])#SENS

performance_testing[3,1]=as.numeric(h2o.specificity(perf,h2o.find_threshold_by_max_metric(perf,"f1"))[[1]])#SPEC

performance_testing[4,1]=as.numeric(h2o.accuracy(perf,h2o.find_threshold_by_max_metric(perf,"f1"))[[1]])#accuracy

performance_testing[5,1]=as.numeric(h2o.precision(perf,h2o.find_threshold_by_max_metric(perf,"f1")[[1]]))#precision

performance_testing[6,1]=as.numeric(h2o.sensitivity(perf,h2o.find_threshold_by_max_metric(perf,"f1"))[[1]])#recall = sens

performance_testing[7,1]=as.numeric(h2o.F1(perf,h2o.find_threshold_by_max_metric(perf,"f1"))[[1]])#F1

performance_testing[8,1]=(performance_testing[2,1]+performance_testing[3,1])/2 #BALANCED ACCURACY

performance_testing_list[[k]]=performance_testing

performance_training_list[[k]]=performance_training

performance_training=matrix( rep( 0, len=3), nrow = 3) #AUC SENS SPECF

performance_testing=matrix( rep( 0, len=8), nrow = 8) # ROC SENS SPEC

}

######################AUC plot_testing

list_test=performance_testing_list

list_train=performance_training_list

ee=lapply(list_test, function(x) x[1,])

output <- do.call(rbind,lapply(ee,matrix,ncol=1,byrow=TRUE))

AUC_mean=apply(output,2,mean)

AUC_mean=data.frame(value=AUC_mean,Algorithm=c('DL'))

#colnames(AUC_mean)=c('DL')

pdf("AUC_mean_testing.pdf")

p<-ggplot(data=AUC_mean, aes(x=Algorithm, y=value)) + geom_bar(stat="identity")

plot(p)

dev.off()

**Validation of methylation results.** Pyrosequencing is a quantitative sequencing method was used to validate the results of the genome-wide methylation analysis. The primers for pyrosequencing were designed manually by randomly choosing two CpG markers based on threshold p-value <0.05 (cg01887804 and cg26340737). We used 1400ng of DNA per reaction and performed bisulfite conversion using Zymo Direct Methylation kit. 42ng of bisulfite treated DNA was used for PCR reaction. Methylation percentage of each CpG locus was determined using a Qiagen (Valencia, CA) Pyromark Q96 ID pyrosequencer and sequencing primer, according to manufacturer’s recommendations. Light intensity was translated as a peak on the Pyrogram from which the percentage CpG methylation was calculated and compared with the results of the Infinium MethylationEPIC CHIP hybridization.

The reverse primer was 5’ biotinylated and the primer sequences used were as follows:

**cg01887804 at TSS200**

Forward primer: GTAAGATGTATTTTGTTATGATTAGTGTG

Reverse primer: 5’ Biotinylation-TACCCAATAAATTAATCAAACATCTCTAAAAT

Sequencing primer: TTTTGTTATGATTAGTGTGT

**cg26340737 at TSS1500 5'UTR**

Forward primer: GTTTTGTAAATTATTTAATATGGGGAGGGT

Reverse primer: 5’ Biotinylation-AAAACAAAACTTAAATCTACCCCTCCCTTT

Sequencing primer: AATGAGTTGGGTTAATATT

**References**

1. Liu Y, Aryee MJ, Padyukov L, Fallin MD, Hesselberg E, Runarsson A *et al.* Epigenome-wide association data implicate DNA methylation as an intermediary of genetic risk in rheumatoid arthritis. *Nat Biotechnol* 2013; **31**(2)**:** 142-147.

2. Chen YA, Lemire M, Choufani S, Butcher DT, Grafodatskaya D, Zanke BW *et al.* Discovery of cross-reactive probes and polymorphic CpGs in the Illumina Infinium HumanMethylation450 microarray. *Epigenetics* 2013; **8**(2)**:** 203-209.

3. Wilhelm-Benartzi CS, Koestler DC, Karagas MR, Flanagan JM, Christensen BC, Kelsey KT *et al.* Review of processing and analysis methods for DNA methylation array data. *Br J Cancer* 2013; **109**(6)**:** 1394-1402.

4. Daca-Roszak P, Pfeifer A, Zebracka-Gala J, Rusinek D, Szybinska A, Jarzab B *et al.* Impact of SNPs on methylation readouts by Illumina Infinium HumanMethylation450 BeadChip Array: implications for comparative population studies. *BMC Genomics* 2015; **16**(1)**:** 1003.

5. Wishart DS. Computational approaches to metabolomics. *Methods Mol Biol* 2010; **593:** 283-313.

6. Chong J, Xia J. MetaboAnalystR: an R package for flexible and reproducible analysis of metabolomics data. *Bioinformatics* 2018; **34**(24)**:** 4313-4314.

7. Jannot AS, Ehret G, Perneger T. P < 5 x 10(-8) has emerged as a standard of statistical significance for genome-wide association studies. *J Clin Epidemiol* 2015; **68**(4)**:** 460-465.

8. Bengio Y. Learning Deep Architectures for AI. *Foundations and Trends® in Machine Learning* 2009; **2**(1)**:** 1-127.

9. Goodfellow I, Bengio Y, Courville A. Deep Learning. *MIT Press* 2016.

10. Huang JH, Xie HL, Yan J, Lu HM, Xu QS, Liang YZ. Using random forest to classify T-cell epitopes based on amino acid properties and molecular features. *Anal Chim Acta* 2013; **804:** 70-75.

11. Cristianini N, Shawe-Taylor J. *An introduction to support Vector Machines: and other kernel-based learning methods*. Cambridge University Press2000, 189pp.

12. Boser BE, Guyon IM, Vapnik VN. *A training algorithm for optimal margin classifiers*. Association for Computing Machinery: Pittsburgh, Pennsylvania, USA, 1992, 144–152pp.

13. Balakrishnama S, Ganapathiraju A. Linear discriminant analysis-a brief tutorial. *Institute for Signal and information Processing 18* 1998**:** 1-8.

14. Candel A, Parmar V, LeDell E, Arora A. *Deep Learning with H2O*, 2018.

15. Kuhn M. Building Predictive Models in R Using the caret Package. *Journal of statistical software* 2008; **28**(5)**:** 1-26.

16. Robin X, Turck N, Hainard A, Tiberti N, Lisacek F, Sanchez J-C *et al.* pROC: an open-source package for R and S+ to analyze and compare ROC curves. *BMC Bioinformatics* 2011; **12**(1)**:** 77.

17. Srivastava N, Hinton G, Krizhevsky A, Sutskever I, Salakhutdinov R. Dropout: a simple way to prevent neural networks from overfitting. *J Mach Learn Res* 2014; **15**(1)**:** 1929-1958.
